# Preparation of Lanthanum-Modified Tea Waste Biochar and Its Adsorption Performance on Fluoride in Water

**DOI:** 10.3390/ma17030766

**Published:** 2024-02-05

**Authors:** Wei Li, Pengcheng Xie, Haiyang Zhou, Huiying Zhao, Bo Yang, Jian Xiong

**Affiliations:** School of Ecology and Environment, Tibet University, Lhasa 850012, China; weili_xzdx@163.com (W.L.); xpc15121632897@163.com (P.X.); zhou1hai5yang7@163.com (H.Z.); huiying7879@163.com (H.Z.); y1161655614@gmail.com (B.Y.)

**Keywords:** tea waste, biochar, lanthanum modification, fluoride, adsorption

## Abstract

In this study, tea waste was used as a raw material, and TBC (tea waste biochar) was prepared by pyrolysis at 700 °C. La(NO_3_)_3_·6H_2_O was used as the modifier to optimize one-way modification; the orthogonal experiment was undertaken to determine the optimal preparation conditions; and La-TBC (lanthanum-modified biochar) was obtained. The key factors for the adsorption of fluoride by La-TBC were investigated by means of batch adsorption experiments, and kinetics and isothermal adsorption experiments were carried out on the adsorption of fluoride in geothermal hot spring water. The adsorption mechanism of fluoride by La-TBC was analyzed via characterization methods such as SEM-EDS (Scanning Electron Microscope and Energy Dispersive Spectrometer), BET (Brunauer–Emmett–Teller), FTIR (Fourier transform infrared), XRD (X-ray diffraction), and so on. The results show that La-TBC had the best adsorption effect on fluoride at pH 7. The process of adsorption of fluoride follows the pseudo-second-order kinetics and Langmuir isothermal model, and the maximum theoretical adsorption quantity was 47.47 mg/g at 80 °C, while the removal rate of fluoride from the actual geothermal hot spring water reached more than 95%. The adsorption process was dominated by the monolayer adsorption of chemicals, and the mechanisms mainly include pore filling, ion exchange, and electrostatic interaction.

## 1. Introduction

Fluorine (F) is an indispensable trace element for the human body, and it is an important factor in maintaining the normal growth and development of human teeth and bones. However, excessive intake of F can jeopardize health by causing osteoporosis, thyroid, liver, and other cellular tissue diseases that cause functional damage [1]. Tibet, China, has abundant geothermal hot spring resources due to its special geological structure, and high concentrations of fluoride represent common harmful substances in hot springs. In the process of geothermal resource development, fluoride sinks into nearby rivers with the hot spring water through runoff, resulting in higher fluoride concentrations in the rivers flowing through the town of Yambajing, Lhasa, Tibet, where there are many geothermal hot springs. It has been shown that the fluoride concentration in the neighboring rivers reaches 23.9 mg/L [2], which is much higher than the upper limit of fluoride concentration in drinking water (1.5 mg/L) stipulated by the World Health Organization (WHO), resulting in fluoride pollution [3]. Tibet functions as an Asian water tower, so fluoride pollution control from geothermal hot springs is of great significance to the water ecological environment and human health.

The current methods for the removal of fluoride mainly include ion exchange and coagulation [4,5], membrane technology [6], electrochemical methods, and adsorption methods [7,8]. Among these, the adsorption method has become one of the most promising technologies due to its low cost, convenience of operation, good treatment effects, and low risk of secondary pollution [9].

Commonly used adsorbents for the treatment of fluoride in water include activated alumina [10], calcite [11], clays [12], metal oxides [13], zeolites [14], and biochar [15]. Among these, biochar is characterized by a large specific surface area, rich surface functional groups, and a stable structure [16]. In addition, biochar is composed of renewable biomass and, thus, is a sustainable adsorbent [17,18].

It is estimated that the daily consumption of tea worldwide is about 18–20 billion cups [19], and China has been a tea-drinking country since ancient times, with a tea culture that goes back a long way. The Chinese consume about 572.6 tons of tea per day [20], especially in Tibet, China, where tea drinking has become an indispensable part of the culture due to the presence of phenolics that have antioxidant and cell-protective effects. Therefore, in the context of such large consumption, a large amount of tea waste is generated, which is mostly disposed of in landfills, incinerated, or composted via a single method, which may create many environmental problems [21]. Tea waste is rich in substances such as lignin and holocellulose, which are rich in functional groups such as carboxyl and hydroxyl [22], meaning that tea waste can be used to prepare biochar, which can not only solve the problem of utilizing tea waste but also help to remove pollutants from water using the prepared biochar material.

Since the surface charge of biochar materials prepared from plant material classes is usually negative, the same as for fluoride, and the electrostatic effect is weak, which prevents the effective adsorption of fluoride [23], the development of effective modification methods to enhance the adsorption efficacy of fluoride is of great significance for biochar research. The main modification methods currently used involve metal modification [24]; one such example shows that the rare earth metal lanthanum (La) has a high affinity for fluoride [25]. However, the high price of lanthanum limits its practical application, so loading lanthanum on inexpensive materials may be an economical way to reduce the amount of lanthanum required while maintaining a high adsorption capacity.

In this study, tea waste was used as a biomass precursor for the preparation of biochar (TBC), which was modified with lanthanum nitrate. The innovation of this study lies in the fact that, compared with previous studies on adsorbent material preparation, one-way experiments, as well as orthogonal experiments, have been used to optimize the preparation conditions and thus enhance the performance of the adsorbents. Lanthanum-modified biochar (La-TBC) was derived from optimal preparation conditions, and batch adsorption experiments were conducted to investigate the effects of La-TBC on fluoride under different experimental conditions, as well as its effects on the adsorption of fluoride in real geothermal hot spring water. The mechanism of adsorption of fluoride by La-TBC was also investigated using the results of SEM-EDS, BET, FTIR, and XRD analyses, which provide technical support for the removal of fluoride from geothermal hot spring water.

## 2. Materials and Methods

### 2.1. Materials and Reagents

The reagents used in this study include lanthanum(III) nitrate hexahydrate (La(NO_3_)_3_·6H_2_O), sodium fluoride (NaF), sodium hydroxide (NaOH), hydrochloric acid (HCI), sodium carbonate (Na_2_CO_3_), sodium bicarbonate (NaHCO_3_), sodium chloride (NaCl), sodium sulfate (Na_2_SO_4_), sodium nitrate (NaNO_3_), and ultrapure water. The ultrapure water was prepared using an ultrapure water system produced by Merck KGaA (Darmstadt, Germany), and the rest of the reagents were analytically pure and purchased from Aladdin Reagent (Shanghai, China).

The brick tea used in this study is one of the most popular products in Tibet, China. A set amount of tea leaves was taken and repeatedly steeped 10 times in ultra-pure water at 90 °C, then dried at 105 °C, crushed through a 100-mesh sieve after cooling, and then steeped again; after the fluoride potential in the supernatant solution was determined to be consistent with the blank signal, the tea waste was dried, sealed, and kept for later use.

### 2.2. Preparation of TBC and La-TBC

Preparation of TBC (tea waste biochar): A certain amount of pre-treated tea dregs was taken from a sealed bag and put into a quartz crucible, and the quartz crucible was put into a tube furnace for pyrolysis in an environment of N_2_. The heating rate was set at 7 °C/min, the pyrolysis temperature was set at 700 °C, and the pyrolysis time was set at 1 h [26]. After pyrolysis was completed, the prepared biochar material was washed with ultrapure water through repeated vacuum filtration; the filtrate was measured to be pH-neutral, and the biochar material was dried at 105 °C for 24 h and then put into a sealed bag for later use, which was recorded as TBC.

Preparation of La-TBC (lanthanum-modified biochar): A certain amount of TBC that had been prepared and preserved was taken out and put into 50 mL centrifugal tubes containing La(NO_3_)_3_·6H_2_O solutions configured in different concentrations, and then put into a water bath thermostatic oscillator for shaking modification. During the modification process, the pH of the modified solution, the solid–liquid ratio of biochar to lanthanum (La) solution (g/mL), the temperature of the modified solution (°C), and the concentration of the modifier (mol/L) were set for one-way experiments in order to optimize the conditions, with the adsorption effect of fluoride as the evaluation criterion. Then, the factors that had a more obvious effect on the adsorption of fluoride were selected for the optimization of conditions in orthogonal experiments, and the adsorption effect of fluoride was also used as the evaluation criterion to obtain optimal preparation conditions. Accordingly, the lanthanum (La)-modified biochar was obtained and marked as La-TBC.

### 2.3. Material Characterization

A scanning electron microscope (SEM-EDS) (SU 8020, Hitachi, Tokyo, Japan) was used to observe the changes in the morphological characteristics of the biochar and the elements contained on the surface. Fourier transform infrared spectroscopy (FTIR) (Great 20, Zhongke Ruijie Technology Company Limited, Tianjing, China) was used to analyze the changes in functional groups of the adsorbent before and after adsorption, and the samples were scanned over a range of 500–4000 cm^−1^ with a resolution of 4 cm^−1^ and 32 scans. A specific surface area and pore size analyzer (BET) (V-sorb 2800TP, Beijing Guoyi Precision Measurement Technology Company Limited, Beijing, China) was used to analyze the changes in specific surface area, pore volume, and pore size before and after the adsorption of the adsorbent, and the samples were pre-treated at 200 °C for 4 h. The measurements were carried out in an environment of liquid nitrogen. The physical phase species contained in the samples were analyzed using an X-ray diffractometer (XRD) (TD-3700, Dandong Tongda Science and Technology Company Limited, Dandong, China), with the angular increment set to 0.08° and the sampling time set to 1 s.

### 2.4. Batch Adsorption Experiments

#### 2.4.1. Single-Factor Effects Experiments

In this part of the experiment, several 50 mL centrifuge tubes were prepared, each containing a volume of 40 mL of fluoride solution with a mass concentration of 19 mg/L. The pH of the solution was adjusted with 0.1 mol/L HCl or 0.1 mol/L NaOH, and a certain amount of adsorbent was added. It was then put into a water-bath shaker for adsorption, with the rotational speed set at 180 rpm/min and the adsorption time set at 8 h. The experimental process was repeated three times, and after the adsorption conditions had been met, the supernatant was filtered using a needle filter with a 0.45 μm membrane, and the fluoride mass concentration of the supernatant was measured using a fluoride ion meter (PXSJ-216F). The fluoride removal rate and the adsorption capacity of the fluoride adsorbent were calculated with the following equations:

(1) $$\eta =\frac{\left(C_{0}-C_{e}\right)}{C_{0}}\times 100\%$$

(2) $$q=\frac{\left(C_{0}-C_{e}\right)\times V}{m}$$

where η is the removal rate of fluoride, %; *C*_0_ and *C_e_* are the mass concentrations of fluoride in the solution before and after adsorption, respectively, mg/L; *q* is the adsorption capacity, mg/g; *V* is the volume of fluoride solution, L; and *m* is the amount of La-TBC added, g.

Adsorption experiments with tea waste and TBC: Several centrifuge tubes containing fluoride solution were prepared; the pH was adjusted to 7.0 ± 0.05, and 0.1 g of tea waste and TBC were added, respectively. Then, the centrifuge tubes containing fluoride solution, which had been dosed with tea waste and TBC, were put into a water-bath thermostatic oscillator at 40 ± 1 °C for adsorption by shaking.

Initial solutions’ pH effect experiments: Using some centrifuge tubes already prepared with fluoride solution, the pH was adjusted (5 to 12 ± 0.05, separated by a gradient of 1), 0.1 g of La-TBC was added, and adsorption was carried out by shaking in a water-bath thermostatic oscillator at an adsorption temperature of 40 ± 1 °C.

Adsorption temperature effect experiment: Some centrifuge tubes were prepared with a fluoride solution; the pH was adjusted to 7.0 ± 0.05, and 0.1 g of La-TBC was added. Then, the centrifuge tubes containing a fluoride solution that had been injected with La-TBC were placed into a water-bath shaker at different temperatures (20, 40, 60, and 80 ± 1 °C) for adsorption by shaking.

Experiment on the effect of adsorbent dosage: Several centrifuge tubes were prepared with fluoride solution, the pH of which was adjusted to 7.0 ± 0.05, and different masses of La-TBC (0.01, 0.02, 0.03, 0.04, 0.05, 0.06, 0.07, 0.08, 0.09, 0.1 g) were added; then, the centrifuge tubes containing fluoride solution with added La-TBC were put into a constant-temperature water-bath oscillator at 40 ± 1 °C for adsorption by shaking. The centrifuge tube was then placed into a water bath shaker at 40 ± 1 °C for shock adsorption.

#### 2.4.2. Kinetic Experiments

Some 50 mL centrifuge tubes were prepared with a volume of 40 mL of fluoride solution at a mass concentration of 19 mg/L. The pH was adjusted to 7.0 ± 0.05, and 0.03 g of La-TBC was injected into the tubes. The ambient temperature of the adsorption environment was set at 40 ± 1 °C, and different adsorption times were set for the determination of the adsorption capacity of La-TBC under these variations.

#### 2.4.3. Isothermal Adsorption Experiments and Thermodynamic Experiments

Some 50 mL centrifuge tubes were prepared, each containing a volume of 40 mL of fluoride solution with different mass concentrations; the pH of the solution was adjusted to 7.0 ± 0.05, 0.03 g of La-TBC was added, and the adsorption time was set to 24 h. Isothermal adsorption was carried out at different adsorption temperatures to determine the amount of fluoride adsorbed by La-TBC at different fluoride concentrations and different adsorption ambient temperatures. An isothermal adsorption model was fitted, and the thermodynamic parameters were determined.

#### 2.4.4. Experiments on the Effect of Coexisting-Ions

In this study, the independent effects of five coexisting anions (Cl^−^, NO_3_^−^, SO_4_^2−^, HCO_3_^−^, and HCO_3_^2−^) on the adsorption of fluoride by La-TBC were evaluated, and the experiments were carried out on a binary system of fluoride with one of the anions, containing 19 mg/L of fluoride paired with 50, 100, or 200 mg/L of each coexisting ion. The dosage of the adsorbent was 0.75 g/L, and the adsorption time was set at 8 h. The adsorption temperature was 40 ± 1 °C, and the initial solution pH was 7.0 ± 0.05.

#### 2.4.5. Adsorption Experiments of Fluoride in Actual Geothermal Hot Spring Water

Fluoride adsorption experiments were carried out by first collecting actual geothermal hot spring water samples. La-TBC was added into a centrifuge tube containing geothermal hot spring water at 2 g/L, and then the centrifuge tube was placed in a constant-temperature water-bath oscillator at 40 °C, operated at 180 rpm/min for 8 h. The filtrate was filtered to determine the fluoride mass concentration, and the removal rate was calculated. The actual hot spring water samples were taken from Yambajing Town, Dangxiong County, Tibet Autonomous Region. A total of five sampling points were set, with the respective samples numbered 1, 2, 3, 4, and 5#. The background concentrations of fluoride in the water samples, as well as the temperature and pH of the water body, are shown in Table 1.

### 2.5. Methods of Analysis

In this study, Excel 2019 was used for all data processing, and Origin 2021pro was used for data visualization to enable graphical processing, model fitting, and statistical analysis.

## 3. Results and Discussion

### 3.1. Results of Adsorption Experiments with Tea Waste and TBC

**Figure 1:**
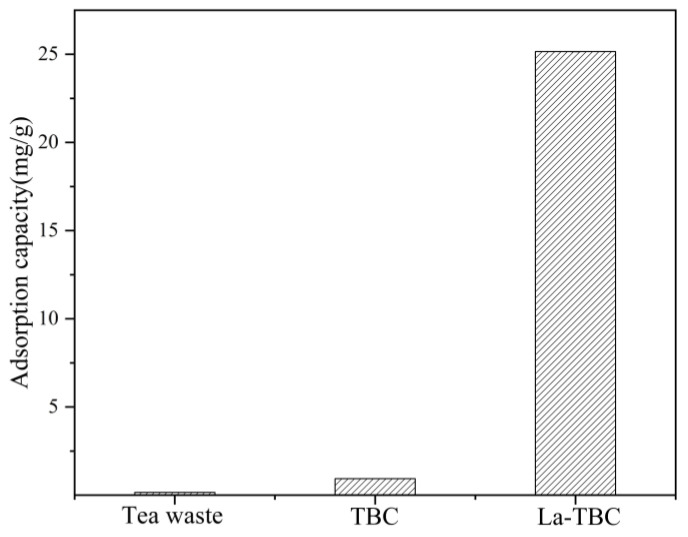
Adsorption of fluoride by tea waste, TBC (tea waste biochar), and La-TBC (lanthanum-modified biochar).

In this experiment, the adsorption capacity of fluoride by tea waste and TBC was investigated and compared with that of fluoride by La-TBC. The experimental results are shown in Figure 1. It can be seen that the adsorption of fluoride by TBC was higher compared to tea waste; the difference was close to 7-fold, but the quantity of adsorption only reached 0.9363 mg/g. However, compared with the capacity for the adsorption of fluoride by La-TBC, the difference was more than 25-fold. Therefore, modifications of TBC were undertaken to improve its fluoride adsorption performance.

### 3.2. Optimization of Lanthanum-Modified Biochar Preparation Conditions

#### 3.2.1. Optimization of Single-Factor Conditions

**Figure 2:**
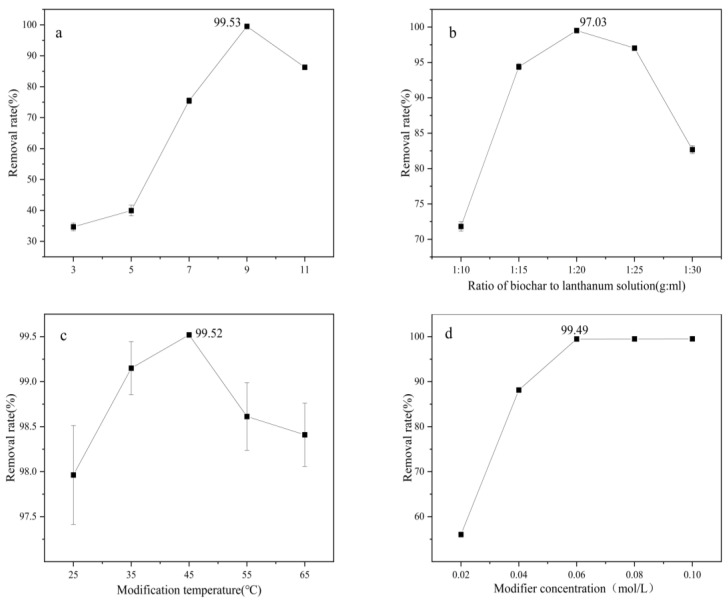
Effect of modified solution pH (**a**), solid–liquid ratio of biochar to lanthanum (La) solution (**b**), modified temperature (**c**), and modifier concentration (**d**) on the removal of fluoride by prepared lanthanum-modified biochar.

To study the effect of the modifier solution’s pH on the fluoride-removal effect of the prepared lanthanum-modified biochar, the modification time was fixed at 10 h. We referred to the experimental results shown in Figure 2a. It can be seen that when the pH of the modified solution was lower than 9.0, the fluoride removal capacity of the prepared modified biochar increased with the increase in pH, but when the pH exceeded 9.0, the fluoride removal rate tended to decrease. The results show that the pH of the preparation process had a significant effect on the fluoride removal effect of the prepared modified biochar. The main reason may be that with the increase in pH, the La^3+^ loaded on the surface of the biochar will generate lanthanide-containing metal oxide precipitates under alkaline conditions that can be loaded onto the tea waste biochar and adsorb fluoride, but when the pH is too high, the metal precipitates form too fast, which may result in the clogging of the pores of some charcoal materials, leading to a decrease in the removal rate of fluoride. Therefore, the pH of the modified solution was set at 9 for subsequent experiments.

To study the effects of the solid–liquid ratio (g/mL; biochar material: lanthanum (La) solution) on the fluoride removal effect of the prepared lanthanum-modified biochar, we referred to the experimental results shown in Figure 2b. With the increase in the solid–liquid ratio, the fluoride removal ability of the modified biochar showed an upward trend, and the fluoride removal rate slightly decreased as the solid–liquid ratio exceeded 1:20 (g/mL). The reason may be that, as the solid–liquid ratio increased, the contact between the biochar material and the lanthanum solution became sufficient, enabling more lanthanum to be loaded onto the biochar to generate more lanthanum-containing metal oxides, which can effectively remove fluoride; however, when the solid–liquid ratio continues to increase, the lanthanum loading of the biochar decreases because of the excess quantity of solution in the modified environment, which, in turn, lowers the fluoride removal effect of the modified biochar. Therefore, a solid–liquid ratio of 1:20 (g/mL) between biochar and lanthanum (La) solution was set as the condition for subsequent experiments.

To study the effect of the modification temperature on the fluoride removal effect of the prepared lanthanum-modified biochar, we used the experimental results shown in Figure 2c. The rate of removal of fluoride by the modified biochar prepared at different temperatures showed an increasing and then decreasing trend, but in general, the difference in fluoride removal effect was not obvious. This difference may be due to the difference in the content of lanthanide-containing metal oxides loaded on the surface of the biochar at different temperatures, which resulted in different removal rates of fluoride. Therefore, a temperature of 45 °C was selected as the modification condition for subsequent experiments.

To study the effect of the modifier concentration on the fluoride removal effect of the prepared lanthanum-modified biochar, we used the experimental results shown in Figure 2d. The fluoride removal effect of modified biochar was obviously improved with an increase in modifier concentration in the range of 0.02~0.06 mol/L. However, when the modifier concentration reached 0.06 mol/L, the fluoride removal rate was close to 100%, which may be because the loading sites on the surface of the biochar were loaded with lanthanum, meaning that each component of the modified charcoal material fully realized its adsorption effect.

#### 3.2.2. Optimization of Orthogonal Experimental Conditions

In this study, based on the optimization of the one-factor experimental conditions, an orthogonal experimental design was used to optimize the preparation process for lanthanum-modified tea waste biochar to investigate its capacity for fluoride adsorption under different conditions, and the results are shown in Table 2.

Using the fluoride adsorption values seen in the above table, the fluoride adsorption capacity of lanthanum-modified tea waste biochar was analyzed for extreme values, and the results are shown in Table 3.

According to the results of the extreme difference R analysis of fluoride adsorption, the degrees of influence can be ranked in descending order, as follows: the pH of the modified solution, the concentration of the modifier, the solid–liquid ratio of the tea residue biochar to the lanthanum solution, and the temperature of the modification (B > D > A > C). Based on the comparison of the K values, the optimal preparation conditions can be identified as follows: a modified solution pH of 9, a modifier concentration of 0.08 mol/L, a modification temperature of 35 °C, and a solid–liquid ratio of the tea waste biochar to lanthanum solution of 1:25 (g/mL) (B_2_D_3_A_1_C_3_). Since this combination was not possible in the orthogonal experiments, the amount of fluoride absorbed by this combination was measured to be 7.58 mg/g after complementary experiments, which is higher than that of the orthogonal experimental group, so the lanthanum-modified biochar prepared via this process was named La-TBC.

### 3.3. Batch Adsorption Experiments

#### 3.3.1. Initial Solution pH Effects

**Figure 3:**
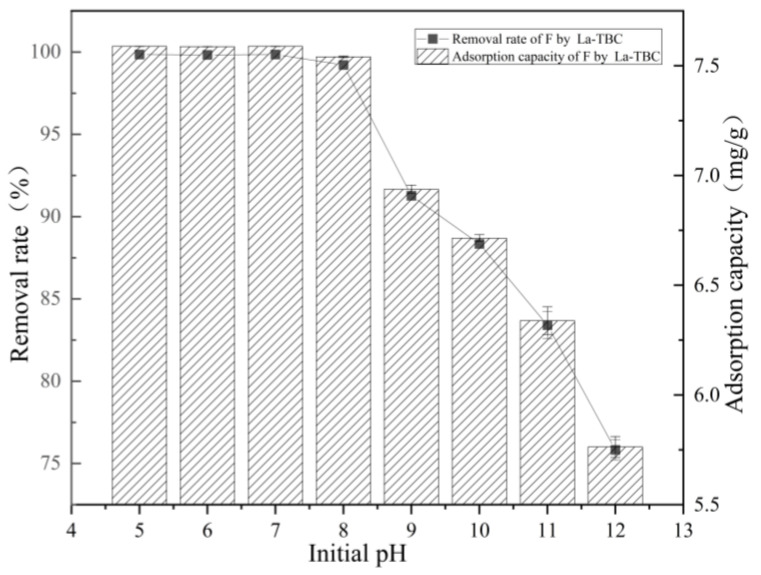
Effect of initial solution pH on adsorption of fluoride by La-TBC.

The initial pH value of the solution directly affects the chemical properties of the adsorbent material and the morphology of the adsorbent in the solution. In this study, the effect of La-TBC on fluoride removal was tested in the pH range of 5.0–12.0. The experimental results are shown in Figure 3; the solution with a pH value of 5.0~7.0 had a good removal effect for fluoride, near total removal. This may be because under acidic conditions, H^+^ and F^−^ generate weakly ionized HF [27], such that the free state of the F^−^ content in the solution is reduced; in addition, the absorption on the active site of the adsorbent material is sufficient, and it can effectively remove fluoride from the solution. However, with the increase in pH value, the effect of the La-TBC material on the removal of fluoride showed a decreasing trend, which may be because the solution was under alkaline conditions and the high concentration of OH^−^ in the solution competed with fluoride adsorption. In addition, the deprotonation of protonated functional groups with the decrease in H^+^ means that the negative charge on the surface of the adsorbent will hinder its electrostatic attraction to fluoride, thus inhibiting its adsorption of fluoride [28,29]; this leads to a decrease in the removal of fluoride. The higher the pH value and the higher the OH^−^ concentration, the more obvious the inhibitory effect on the removal of fluoride by the material. In general, the overall adsorption and removal rates of the materials reached more than 75.2%, indicating that La-TBC can achieve the effective removal of fluoride in a wide range of pH values.

#### 3.3.2. Effect of Adsorption Temperature

**Figure 4:**
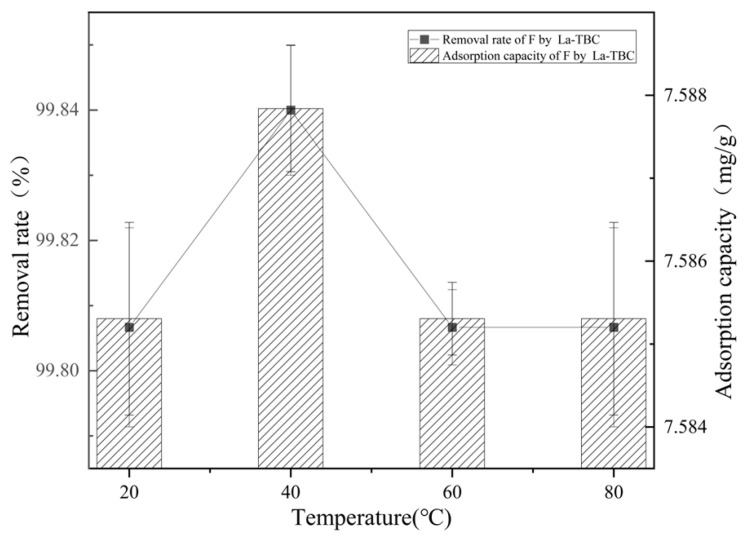
Effect of solution temperature on the adsorption of fluoride by La-TBC.

Actual geothermal hot spring water environments usually have different temperatures, so in this study, we tested the fluoride removal effect of La-TBC at adsorption temperatures of 20, 40, 60, and 80 °C. The experimental results are shown in Figure 4. The material showed a good effect of removing fluoride under different temperature conditions, with a removal rate above 99.8%, which indicates that the removal effect of the material was stable under different temperature conditions. The adsorption of fluoride under high-temperature conditions was stable, and no desorption phenomenon occurred. This means that this process can be adapted to different environments and has broad practical applicability.

#### 3.3.3. Effect of Adsorbent Dosage

**Figure 5:**
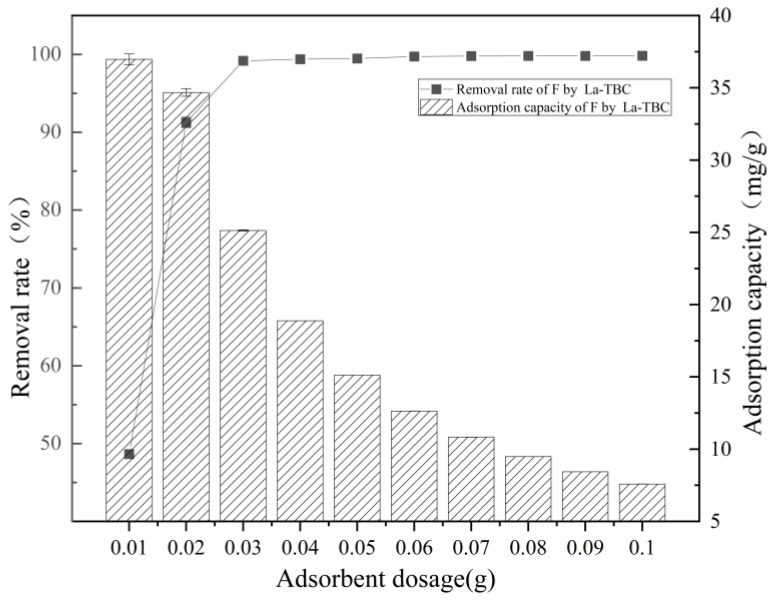
Effect of adsorbent dosage on the adsorption of fluoride by La-TBC.

The degree of adsorbent dosing can affect the number of active adsorption sites on the material, which, in turn, affects the adsorption effect of fluoride. In this study, the adsorption capacity and removal rate of fluoride by La-TBC were tested for 0.01, 0.02, 0.03, 0.04, 0.05, 0.06, 0.07, 0.08, 0.09, and 0.1 g of adsorbent dosing. The experimental results are shown in Figure 5, which show that as the adsorbent dosage increased from 0.01 g to 0.03 g, the removal of fluoride increased significantly (from 49.19% to 99.32%), but when the adsorbent dosage continued to increase from 0.03 g to 0.1 g, the increase in the removal of fluoride was not significant. On the contrary, the adsorption capacity of fluoride decreased with the increase in La-TBC dosage (from 37.38 mg/g to 7.59 mg/g). The reason for this may be that the amount of fluoride in the solution held a constant value, and when the La-TBC dosage increased, the number of idle adsorption active sites also increased. Moreover, when too much adsorbent is dosed, there is competition between the adsorbents for fluoride adsorption, which can slightly reduce the adsorption efficiency [30].

### 3.4. Kinetic Studies

**Figure 6:**
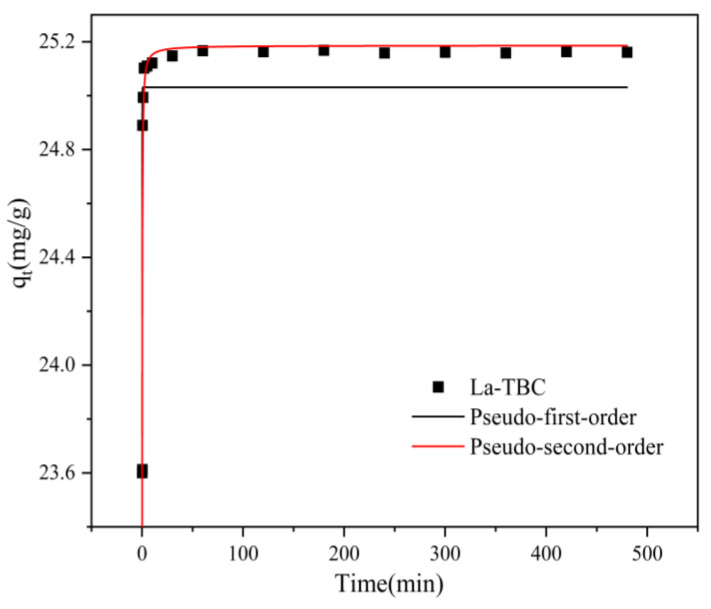
The kinetic curve of fluoride adsorption by La-TBC.

We next sought to evaluate the rate of fluoride adsorption on La-TBC and to understand its mechanism. In this study, the kinetic experimental data were fitted using pseudo-first-order kinetic equation fitting (Equation (3)) and the pseudo-second-order kinetic equation (Equation (4)). The results of the two kinetic models are shown in Figure 6, and the model fit parameters are shown in Table 4.

(3) $$q_{t}=q_{e}\left(1-e^{-k_{1}t}\right)$$

(4) $$q_{t}=\frac{{q_{e}}^{2}k_{2}t}{1+q_{e}k_{2}t}$$

Here, *q_t_* and *q_e_* are the adsorption capacity at time *t* and equilibrium, mg/g; *t* is the adsorption time, min; *k*_1_ is the pseudo-first-order kinetic constant, min^−1^; and *k*_2_ is the pseudo-second-order kinetic constant, g/(mg·min).

**Table 4:** Parameters fitted to the kinetic model of fluoride adsorption by La-TBC.

Pseudo-First-Order				Pseudo-Second-Order		
*q*_*e*,*exp*_/(mg/g)	*k*_1_/(min^−1^)	*q*_*e*,*cal*_/(mg/g)	*R* ^2^	*k*_2_/(g/(mg·min))	*q*_*e*,*cal*_/(mg/g)	*R* ^2^
25.16	15.03	25.03	0.345	2.88	25.19	0.807

As can be seen from the figure, the fitted results for the adsorption of fluoride by La-TBC are more consistent with the pseudo-second-order kinetic model. Comparing the fitting correlation coefficients between the pseudo-first-order kinetic and pseudo-second-order kinetic models, we see that the pseudo-second-order kinetic model has a higher *R^2^* (0.807), and the difference between its theoretical adsorption capacity, *q*_*e*,*cal*_, and the experimental value, *q*_*e*,*exp*_, is smaller, which suggests that the adsorption process of fluoride ions by La-TBC is dominated by chemical adsorption [31].

### 3.5. Isothermal Adsorption Studies

**Figure 7:**
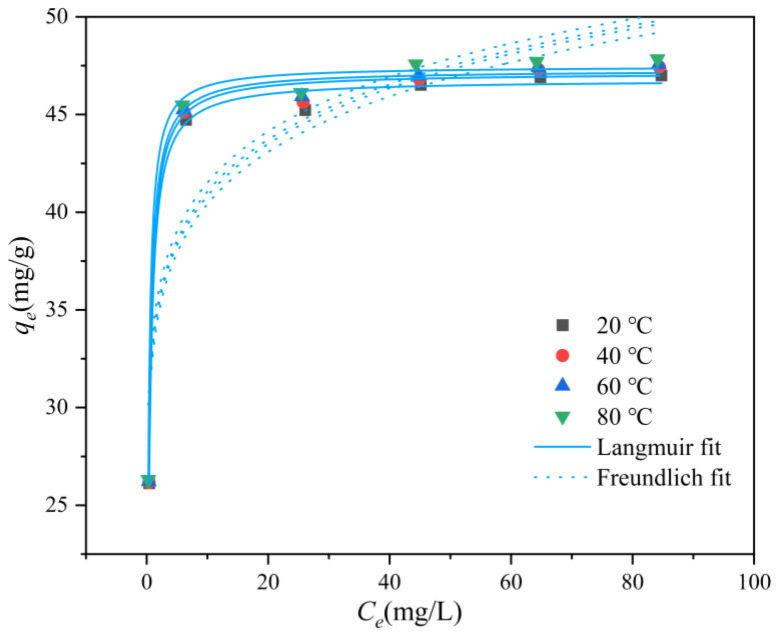
Isothermal adsorption model fit curve of fluoride on La-TBC.

The adsorption isotherm describes the adsorption capacity of the adsorbent at different initial concentration levels. In this study, Langmuir (Equation (5)) and Freundlich (Equation (6)) models were used to investigate the isothermal adsorption process of fluoride by La-TBC at different temperatures. The fitted curves and parameters of fluoride adsorption by La-TBC at different temperatures are shown in Figure 7 and Table 5.

(5) $$q_{e}=\frac{q_{m}K_{L}C_{e}}{1+K_{L}C_{e}}$$

(6) $$q_{e}=K_{F}{C_{e}}^{1/n}$$

Here, *q_m_* is the maximum monolayer adsorption capacity, mg/g; *K_L_* is the Langmuir adsorption equilibrium constant, L/mg; *K_F_* is the Freundlich adsorption equilibrium constant, mg/g; and n is the Freundlich isothermal constant for the strength of adsorption.

**Table 5:** Isothermal adsorption model fitting parameters of fluoride on La-TBC.

T (°C)	Langmuir Isotherm			Freundlich Isotherm		
	*q_m_* (mg/g)	*K_L_*/(L/mg)	*R* ^2^	*K_F_* (mg/g)	*n*	*R* ^2^
20	46.79	3.143	0.995	32.7	10.87	0.786
40	47.14	3.358	0.995	33.08	10.99	0.783
60	47.28	3.638	0.996	33.33	11.11	0.786
80	47.47	4.631	0.994	33.87	11.36	0.802

From the isothermal adsorption model fitting curves, it can be seen that the fluoride adsorption capacity of La-TBC at different temperatures increased with the increase in the initial concentration of the solution and eventually leveled off, and the adsorption amount increased from 26.31 to 47.85 mg/g. Compared to the performance of fluoride adsorption from groundwater using lanthanum-modified pomelo peel biochar (19.86 mg/g), studied by Wang et al. [32], the adsorption of fluoride by La-TBC was elevated by nearly 1.5 times. The Langmuir model fitting coefficients *R*^2^ were higher than those of the Freundlich model (Table 4). In addition, the theoretical maximum adsorption capacity obtained from the fitting of the Langmuir equation is closer to the real experimental result (47.84 mg/g), which indicates that the Langmuir model is more descriptive of the adsorption behavior of La-TBC, which we can thus infer follows a homogeneous monolayer adsorption process [33]. According to the results of the Freundlich model, the n value of La-TBC is greater than 1 at different temperatures, indicating that the adsorption process is easily completed. According to the above experimental results, it can be concluded that La-TBC has good adsorption properties.

### 3.6. Adsorption Thermodynamic Studies

In this study, the adsorption mechanism of fluoride by La-TBC was investigated via adsorption thermodynamics. Three thermodynamic parameters were investigated: standard Gibbs free energy (Δ*G*^0^), standard enthalpy (Δ*H*^0^), and standard entropy (Δ*S*^0^) calculated from Equations (7)–(9). We first calculated *K_0_* at the first concentration gradient for each temperature, according to Equation (7), then found the Gibbs free energy change of the adsorption reaction at different temperatures Δ*G*^0^ according to Equation (8). The enthalpy change Δ*H*_0_ and entropy change Δ*S*_0_ of the adsorption reaction could then be derived from Equation (9) by plotting 1/T through ln*K_b_*, and through the slope and the intercept. The results are shown in Table 6.

(7) $$K_{0}=\frac{q_{e}}{C_{e}}$$

(8) $$\Delta G^{0}=-RT\ln K_{0}$$

(9) $$\ln K_{0}=\frac{\Delta S^{0}}{R}-\frac{\Delta H^{0}}{RT}$$

Here, *T* is the reaction temperature, K; *K*_0_ is the adsorption equilibrium constant, L/g; and *R* is the ideal gas constant, which takes the value of 8.314 × 10^−3^ kJ·(mol·K)^−1^.

**Table 6:** Thermodynamic parameters of fluoride adsorption by La-TBC.

Δ*G*^0^ (kJ·mol^−1^)				Δ*S*^0^ (kJ·(mol·k)^−1^)	Δ*H*^0^ (kJ·mol^−1^)
20 °C	40 °C	60 °C	80 °C		
−10.171	−11.078	−12.023	−13.477	0.0536	5.652

As can be seen from Table 6, Δ*H*_0_ > 0 indicates that the process of fluoride adsorption by La-TBC is a heat-absorbing reaction. At all temperature conditions, Δ*G*^0^ was negative, indicating that the adsorption of fluoride by La-TBC is a spontaneous process, and its value decreases with an increasing temperature, suggesting that an increasing temperature is more favorable for spontaneity [34]. Δ*S*_0_ > 0 indicates that the disorder of the adsorbent solid/liquid interface increases as the adsorption reaction proceeds [35], and the adsorbent’s surface structure is affected.

### 3.7. Co-Existing Ion Impact Studies

**Figure 8:**
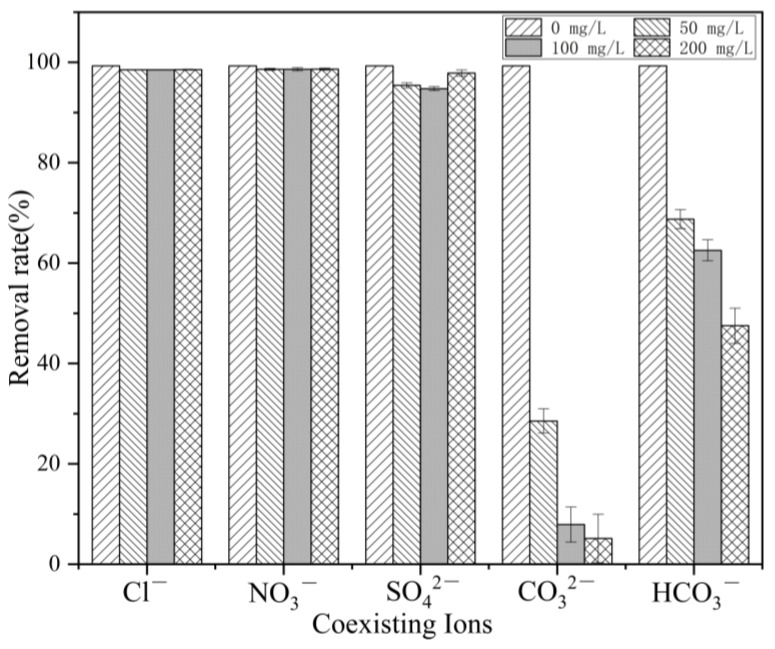
Effect of coexisting ions on fluoride removal.

To test the feasibility of applying La-TBC in a real geothermal hot spring water environment, this study conducted experiments on the effects of several anions (Cl^−^, NO_3_^−^, CO_3_^2−^, SO_4_^2−^, and HCO_3_^−^) on the removal of fluoride at three levels of concentration (50.0, 100.0, and 200.0 mg/L). The experimental results are shown in Figure 8; the presence of NO_3_^−^ and Cl^−^ had almost no effect on the adsorption of fluoride, and the removal rate remained above 98.5%. On the other hand, the presence of CO_3_^2−^, SO_4_^2−^ and HCO_3_^−^ had different degrees of influence on the adsorption of fluoride—when the concentrations of HCO_3_^−^ and CO_3_^2−^ increased to 300 mg/L, the removal rate of fluoride by La-TBC dropped to 5.14%. The reason for this may be that when CO_3_^2−^ and HCO_3_^−^ are added to fluoride-containing solutions, a hydrolysis reaction occurs, causing the solution’s pH to rise and become more alkaline, resulting in a drastic decrease in fluoride removal [36].

### 3.8. Actual Geothermal Hot Spring Water Adsorption Experiments

**Figure 9:**
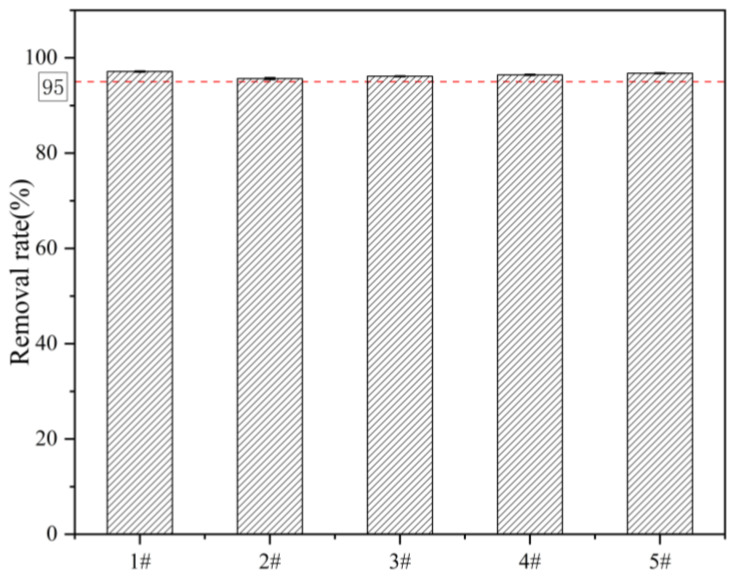
Effect of La-TBC on the removal of moderate fluoride in real geothermal hot spring water.

Adsorption experiments were carried out on the actual geothermal hot spring water to study the potential of La-TBC for use in practical applications, and the experimental results are shown in Figure 9. It can be seen that the fluoride removal rate of La-TBC in five geothermal water samples reached more than 95%, but in the batch adsorption experiments, the removal rate reached 99.32% with the same adsorption time and adsorption temperature; the dosage was only half that in the adsorption experiments of actual geothermal hot springs, and the initial fluoride solution was 19 mg/L. The reason for this phenomenon may be that there are different concentrations of HCO_3_^−^, HCO_3_^2−^, SO_4_^2−^, and microorganisms competing for adsorption in geothermal hot spring water, and the adsorption effect is inhibited under alkaline conditions, so the removal rate of fluoride in actual geothermal hot spring water can be improved by increasing the dosage of the adsorbent.

### 3.9. Adsorption Mechanism

#### 3.9.1. SEM-EDS and BET Analysis

**Figure 10:**
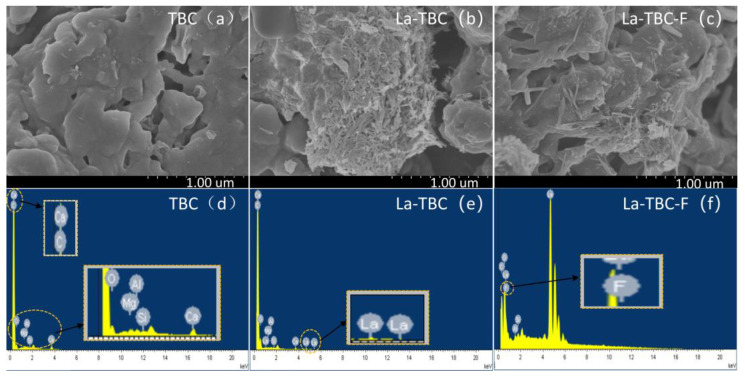
SEM images of TBC (**a**), La-TBC (**b**), and La-TBC-F (**c**), and EDS energy spectra of TBC (**d**), La-TBC (**e**), and La-TBC-F (**f**).

At the same magnification scale (1 μm), the scanning electron microscope (SEM) plots of TBC and La-TBC with La-TBC-F (material after adsorption of fluoride ions by La-TBC), as well as the EDS energy spectra, are shown in Figure 10. As can be seen in Figure 10a, the TBC surface is smoother and contains abundant pores. Figure 10b shows that La-TBC exhibited a rougher surface and many brighter regions, with more columnar material appearing, and it was found that the pore structure on the surface of the material was reduced, which may have been because the lanthanide (La)-containing material was sufficiently loaded to fill the pores [37]. When the La-TBC adsorbed fluoride (Figure 10c), the columnar material on the surface of La-TBC-F decreased, and a more obvious pore structure reappeared, which may have been due to the reaction of the lanthanum (La)-containing material on the surface of La-TBC with fluoride during fluoride adsorption. From Figure 10d, it can be seen that the elemental composition of the surface of the original biochar TBC mainly comprised C, Si, Ga, Mg, and Al, indicating that the main components of the material are inorganic salts, whereas, from Figure 10e, it can be inferred that La appeared on the surface of the material, indicating that the lanthanum was successfully loaded onto the surface of the TBC after modification in pursuit of La-TBC. When La-TBC was cast into the fluoride-containing solution used for the adsorption reaction, a new element (F) appeared on the surface of the material, yielding La-TBC-F after the adsorption of fluoride (see Figure 10f). Moreover, the percentage concentrations of elements in the three materials TBC, La-TBC, and La-TBC-F show that lanthanum was successfully loaded onto the surface of TBC, and the fluoride in the solution was successfully adsorbed by the La-TBC (Table 7).

The specific surface areas and pore sizes of TBC, La-TBC, and La-TBC-F were determined, and the results are shown in Table 8. From the results, we see that the specific surface area of TBC reached 110.9 m^2^/g, thus providing sufficient loading sites for lanthanum (La) modification, and when TBC was modified by lanthanum, the specific surface area of La-TBC was significantly decreased (down to 5.2 m^2^/g), and the microporous pore volume also decreased with a quantum variation. The reason may be related to the loading of lanthanum (La) during the modification process [38,39], which is consistent with the results observed in the SEM images. However, the average pore size of La-TBC after modification increased to 244.4 nm, which is more than 100 times greater than the average pore size of TBC (2.2 nm), and thus may be attributed to the fact that some of the thinner pore wall structures in the TBC collapsed during the lanthanum (La) modification process, increasing the average pore size. When fluoride was adsorbed, the specific surface area and microporous pore volume of La-TBC-F increased, and the average pore diameter and total pore volume decreased, which could be attributed to the reaction of lanthanide (La)-containing substances on the surface of La-TBC with fluoride. This would have generated new pore structures, increasing the specific surface area that was involved in pore-filling during the adsorption process.

#### 3.9.2. FTIR Analysis

**Figure 11:**
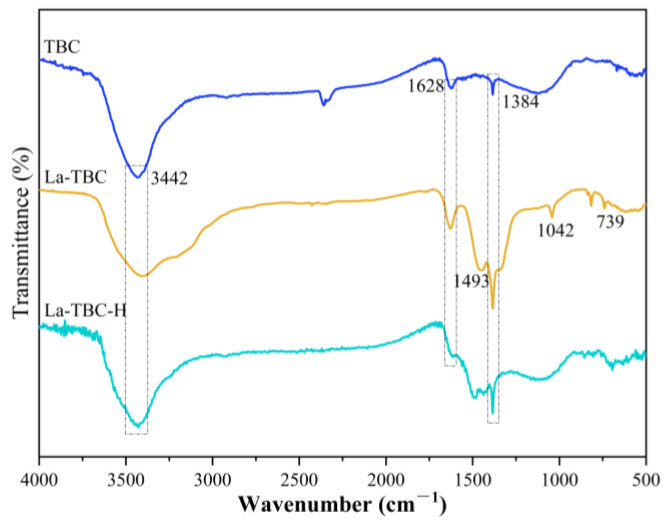
FTIR spectra of TBC, La-TBC, and La-TBC-F.

Fourier transform infrared spectroscopy (FTIR) revealed the types of functional groups on the surface of the biochar material, as well as the changes. Figure 11 shows the FTIR spectra of TBC, La-TBC, and La-TBC-F. Among these, the characteristic peaks of the three biochar materials that appeared at wave number 3442 cm^−1^ were mainly due to the O–H stretching vibration in the H_2_O on the surface of the materials [40]; the characteristic peaks appearing at wave number of 1628 cm^−1^ were caused by the vibration of C=O and C=C, which indicates that the carbon composition of the materials was complex [41]; the characteristic peaks appearing at 1384 cm^−1^ were caused by the vibration of −NO_3_, and the intensity of the characteristic peaks was increased after modification, which implies the presence of nitric oxides, such as nitrate, in the materials [42]. Comparing the TBC spectra, we see that those for La-TBC showed new characteristic absorption peaks at wave numbers of 1493 versus 1042 cm^−1^, which can be attributed to the reaction of La(OH)_3_ with the CO_2_ in air to form carbonate groups [43], and the peak at 739 cm^−1^ can be attributed to the La–OH vibration [32], indicating that lanthanum (La) has been successfully loaded. After the fluoride removal reaction, the intensity of the peak at 1384 cm^−1^ was weakened, suggesting that an ion exchange reaction may have occurred between the nitrate ion and the fluoride ion; moreover, the characteristic absorption peaks at 1042 and 739 cm^−1^ disappeared, suggesting that there was an electrostatic interaction between the fluoride and the La-TBC surface.

#### 3.9.3. XRD Analysis

**Figure 12:**
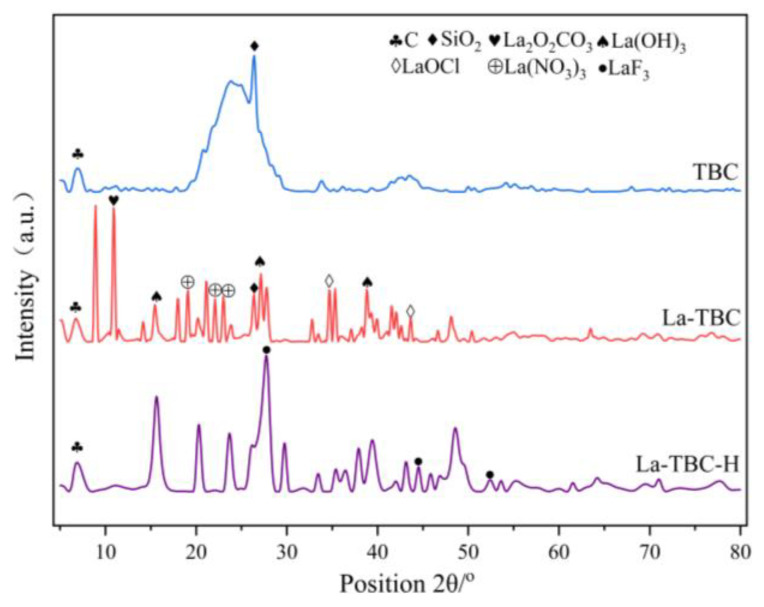
XRD spectra of TBC, La-TBC, and La-TBC-F.

X-ray diffraction (XRD) spectroscopy can be used to determine the type of physical phase contained in the biochar material. Figure 12 shows the XRD patterns of TBC, La-TBC, and La-TBC-F. In the XRD spectrum of TBC, the characteristic diffraction peaks of C (PDF Card:97-018-1083) as well as SiO_2_ (PDF Card:97-003-9830) appeared, which indicates that TBC is a carbon-containing material enriched with SiO_2_. When modified with lanthanum (La), the XRD patterns of La-TBC revealed a series of La_2_O_2_CO_3_ (PDF Card:00-037-0804), La(OH)_3_ (PDF Card:97-016-7480), La(NO_3_)_3_ (PDF Card:00-024-1112), and LaOCl (PDF Card:97-004-0297). Lanthanum (La)-containing oxides with characteristic diffraction peaks [44], indicating that lanthanum (La) was successfully loaded onto the surfaces of TBCs after modification, and various lanthanum (La)-containing substances were generated. After the adsorption of fluoride, the characteristic diffraction peaks of La_2_O_2_CO_3_, La(NO_3_)_3_, and LaOCl could no longer be seen on the XRD pattern of La-TBC-F, which indicates that these substances took part in the fluoride removal reaction during the adsorption process. The diffraction peaks of LaF_3_ (PDF Card: 97-000-0003) also appeared, suggesting that the fluoride was adsorbed successfully by La-TBC. The XRD pattern analysis suggests that the process of the adsorption of fluoride by La-TBC may involve an ion exchange reaction, which is consistent with the results of the FTIR analysis.

**Figure 13:**
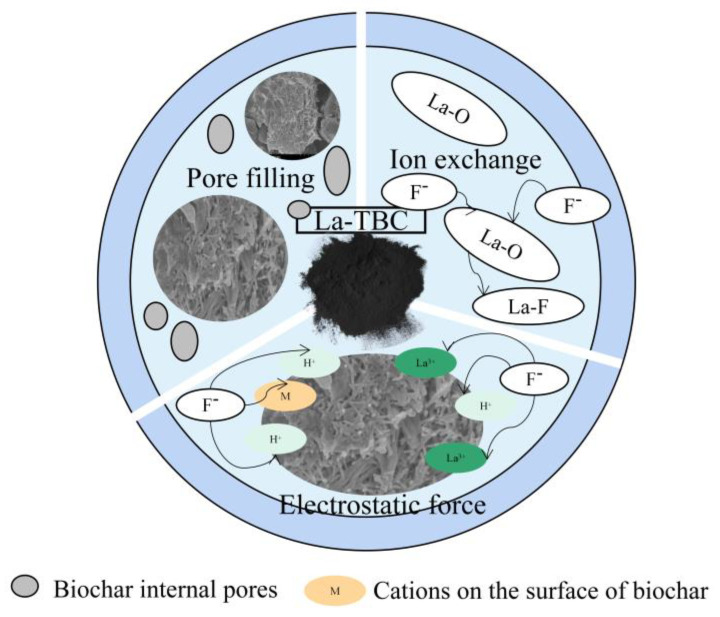
Mechanism of fluoride adsorption by La-TBC.

In summary, the results of SEM, EDS, BET, FTIR, and XRD for TBC, La-TBC, and La-TBC-F show that the lanthanide-containing substances on the surface of La-TBC played a significant role in the adsorption of fluoride from an aqueous solution and that the surface structure had a specific influence on the adsorption. The mechanisms involved in the adsorption process mainly include the filling of the pores on the surface of La-TBC with fluoride, the ion exchange reaction between the lanthanide-containing (La) oxides on the surface of La-TBC and fluoride ions, and electrostatic interactions between the cationic charges on the surface of La-TBC and fluoride ions (see Figure 13).

## 4. Conclusions

(1) Tea waste biochar (TBC) was obtained via the pyrolysis of tea waste as a raw material at 700 °C for 1 h. The one-factor modification and orthogonal optimization of modification using La(NO_3_)_3_·6H_2_O were carried out, and the optimal conditions for preparation were determined: the pH of the modification solution was 9, the concentration of the modifying agent was 0.08 mol/L, the temperature of modification was 35 °C, and the solid–liquid ratio of the tea waste biochar to lanthanum solution was 1:25 (g/mL). Lanthanum-modified biochar (La-TBC) was thus obtained.
(2) When the initial fluoride mass concentration was 19 mg/L and the dosage of La-TBC was 0.75 g/L, the removal rate of fluoride by La-TBC reached 99.32% under the conditions of pH 7, an adsorption time of 8 h, and an adsorption temperature of 40 °C. Under the same adsorption conditions, the adsorption rates of SO_4_^2−^, HCO_3_^−^, and CO_3_^2−^ showed different effects on fluoride, with CO_3_^2−^ having the greatest effect, while Cl^−^ and NO_3_^−^ had almost no effect.
(3) The adsorption of fluoride by La-TBC was more consistent with the pseudo-second-order model and the Langmuir isothermal model. The maximum theoretical adsorption capacity of La-TBC was 47.47 mg/g at 80 °C, which is nearly 50 times higher than the adsorption performance of TBC in relation to fluoride (0.9363 mg/g). The adsorption process was a chemisorption-dominated monolayer adsorption process with spontaneous heat absorption. The adsorption mechanisms mainly included pore filling, ion exchange, and electrostatic interactions.
(4) La-TBC had a good effect on fluoride removal in actual geothermal hot spring water (removal rate of more than 95%), but HCO_3_^−^, HCO_3_^2−^, SO_4_^2−^, and microorganisms in the water body will compete for adsorption, which affects the removal effect, so the fluoride removal rate in geothermal water can be increased by increasing the dosage of the adsorbent.

## Figures and Tables

**Table 1:** Background values of real geothermal hot spring water samples.

Number	Fluoride Concentration (mg/L)	pH	Temperature (°C)
1#	5.44	7.48	46
2#	10.32	9.08	84
3#	11.49	9.17	83
4#	8.76	8.69	81
5#	11.45	8.76	82

**Table 2:** Results of orthogonal experiments of lanthanum-modified tea waste biochar.

Serial Number	Modification Temperature (°C)	pH	Ratio of Biochar to Lanthanum Solution (g/mL)	Modifier Concentration (mol/L)	Fluoride Adsorption Capacity (mg/g)
	A	B	C	D	
1	35	7	1:15	0.04	4.95
2	35	9	1:20	0.06	7.56
3	35	11	1:25	0.08	7.04
4	45	7	1:20	0.08	5.51
5	45	9	1:25	0.04	6.5
6	45	11	1:15	0.06	6.05
7	55	7	1:25	0.06	5.32
8	55	9	1:15	0.08	7.18
9	55	11	1:20	0.04	5.72

**Table 3:** An extreme value analysis of lanthanum-modified tea waste biochar.

Fluoride Adsorption Capacity (mg/g)				
	A	B	C	D
**k_1_**	6.517	5.26	6.06	5.723
**k_2_**	6.02	7.08	6.263	6.31
**k_3_**	6.073	6.270	6.287	6.577
**R**	0.497	1.82	0.227	0.854

**Table 7:** Elemental analysis table for TBC, La-TBC, and La-TBC-F.

Materials	TBC	La-TBC	La-TBC-F
C	89.42	83.42	49.48
O	10.04	16.25	32.89
Mg	0.13	0.06	-
Al	0.10	0.05	0.43
Si	0.08	0.06	0.64
Ca	0.23	0.10	-
La	-	0.07	10.77
F	-	-	5.79

**Table 8:** Specific surface area, average pore size, and total pore volume of TBC, La-TBC, and La-TBC-F.

Materials	BET (m^2^/g)	Micropore Area (m^2^/g)	External Surface Area (m^2^/g)	Average Pore Diameter (nm)	Total Pore Volume (cm^3^/g)	Micropore Volume (cm^3^/g)
TBC	110.9	90.8	20.1	2.2	0.1	3 × 10^−2^
La-TBC	5.2	1.2	4.0	244.4	0.3	5 × 10^−4^
La-TBC-F	9.5	2.0	7.5	30.5	0.1	8 × 10^−4^

## Data Availability

Data are contained within the article.

## References

[1] Ozsvath D.L. (2008). Fluoride and environmental health: A review. Rev. Environ. Sci. Bio/Technol..

[2] Wang Y., Cheng H. (2023). Environmental fate and ecological impact of the potentially toxic elements from the geothermal springs. Environ. Geochem. Health.

[3] Lacson C.F.Z., Lu M.-C., Huang Y.-H. (2021). Fluoride-containing water: A global perspective and a pursuit to sustainable water defluoridation management—An overview. J. Clean. Prod..

[4] Zarrabi M., Samadi M.T., Sepehr M.N., Ramhormozi S.M., Azizian S., Amrane A. (2014). Removal of Fluoride Ions by Ion Exchange Resin: Kinetic and Equilibrium Studies. Environ. Eng. Manag. J..

[5] Ozairi N., Mousavi S.A., Samadi M.T., Seidmohammadi A., Nayeri D. (2020). Removal of fluoride from water using coagulation-flocculation process: A comparative study. Desalination Water Treat..

[6] Damtie M.M., Woo Y.C., Kim B., Hailemariam R.H., Park K.-D., Shon H.K., Park C., Choi J.-S. (2019). Removal of fluoride in membrane-based water and wastewater treatment technologies: Performance review. J. Environ. Manag..

[7] Pulkka S., Martikainen M., Bhatnagar A., Sillanpää M. (2014). Electrochemical methods for the removal of anionic contaminants from water—A review. Sep. Purif. Technol..

[8] Habuda-Stanić M., Ravančić M., Flanagan A. (2014). A Review on Adsorption of Fluoride from Aqueous Solution. Materials.

[9] Bhan C., Singh J., Sharma Y.C., Koduru J.R. (2022). Synthesis of lanthanum-modified clay soil-based adsorbent for the fluoride removal from an aqueous solution and groundwater through batch and column process: Mechanism and kinetics. Environ. Earth Sci..

[10] Craig L., Stillings L.L., Decker D.L., Thomas J.M. (2015). Comparing activated alumina with indigenous laterite and bauxite as potential sorbents for removing fluoride from drinking water in Ghana. Appl. Geochem..

[11] Turner B.D., Binning P., Stipp S. (2005). Fluoride removal by calcite: Evidence for fluorite precipitation and surface adsorption. Environ. Sci. Technol..

[12] Uddin M.K., Ahmed S.S., Naushad M. (2019). A mini update on fluoride adsorption from aqueous medium using clay materials. Desalination Water Treat..

[13] Adak M.K., Sen A., Mukherjee A., Sen S., Dhak D. (2017). Removal of fluoride from drinking water using highly efficient nano-adsorbent, Al(III)-Fe(III)-La(III) trimetallic oxide prepared by chemical route. J. Alloys Compd..

[14] Gómez-Hortigüela L., Pérez-Pariente J., García R., Chebude Y., Díaz I. (2013). Natural zeolites from Ethiopia for elimination of fluoride from drinking water. Sep. Purif. Technol..

[15] Sadhu M., Bhattacharya P., Vithanage M., Padmaja Sudhakar P. (2021). Adsorptive removal of fluoride using biochar—A potential application in drinking water treatment. Sep. Purif. Technol..

[16] Weber K., Quicker P. (2018). Properties of biochar. Fuel.

[17] Rao R.A.K., Ikram S., Uddin M.K. (2014). Removal of Cr(VI) from aqueous solution on seeds of *Artimisia absinthium* (novel plant material). Desalination Water Treat..

[18] Uddin M.K., Abd Malek N.N., Jawad A.H., Sabar S. (2022). Pyrolysis of rubber seed pericarp biomass treated with sulfuric acid for the adsorption of crystal violet and methylene green dyes: An optimized process. Int. J. Phytoremediat..

[19] Hussain S., Anjali K.P., Hassan S.T., Dwivedi P.B. (2018). Waste tea as a novel adsorbent: A review. Appl. Water Sci..

[20] Pan S.-Y., Nie Q., Tai H.-C., Song X.-L., Tong Y.-F., Zhang L.-J.-F., Wu X.-W., Lin Z.-H., Zhang Y.-Y., Ye D.-Y. (2022). Tea and tea drinking: China’s outstanding contributions to the mankind. Chin. Med..

[21] Debnath B., Haldar D., Purkait M.K. (2021). Potential and sustainable utilization of tea waste: A review on present status and future trends. J. Environ. Chem. Eng..

[22] Kabir M.M., Mouna S.S.P., Akter S., Khandaker S., Didar-ul-Alam M., Bahadur N.M., Mohinuzzaman M., Islam M.A., Shenashen M.A. (2021). Tea waste based natural adsorbent for toxic pollutant removal from waste samples. J. Mol. Liq..

[23] Isaac R., Siddiqui S., Aldosari O.F., Kashif Uddin M. (2023). Magnetic biochar derived from *Juglans regia* for the adsorption of Cu^2+^ and Ni^2+^: Characterization, modelling, optimization, and cost analysis. J. Saudi Chem. Soc..

[24] Lee H.-S., Shin H.-S. (2021). Competitive adsorption of heavy metals onto modified biochars: Comparison of biochar properties and modification methods. J. Environ. Manag..

[25] Habibi N., Rouhi P., Ramavandi B. (2018). Modification of *Tamarix hispida* Biochar by Lanthanum Chloride for Enhanced Fluoride Adsorption from Synthetic and Real Wastewater. Environ. Prog. Sustain. Energy.

[26] Peiris C., Nayanathara O., Navarathna C.M., Jayawardhana Y., Nawalage S., Burk G., Karunanayake A.G., Madduri S.B., Vithanage M., Kaumal M.N. (2019). The influence of three acid modifications on the physicochemical characteristics of tea-waste biochar pyrolyzed at different temperatures: A comparative study. RSC Adv..

[27] Merodio-Morales E.E., Reynel-Ávila H.E., Mendoza-Castillo D.I., Duran-Valle C.J., Bonilla-Petriciolet A. (2019). Lanthanum- and cerium-based functionalization of chars and activated carbons for the adsorption of fluoride and arsenic ions. Int. J. Environ. Sci. Technol..

[28] Prabhu S.M., Elanchezhiyan S.S., Lee G., Meenakshi S. (2016). Defluoridation of water by Tea-bag model using La^3+^ modified synthetic resin@chitosan biocomposite. Int. J. Biol. Macromol..

[29] Roy S., Sengupta S., Manna S., Das P. (2018). Chemically reduced tea waste biochar and its application in treatment of fluoride containing wastewater: Batch and optimization using response surface methodology. Process Saf. Environ. Prot..

[30] Dehghani M.H., Gholami S., Karri R.R., Lima E.C., Mahvi A.H., Nazmara S., Fazlzadeh M. (2021). Process modeling, characterization, optimization, and mechanisms of fluoride adsorption using magnetic agro-based adsorbent. J. Environ. Manag..

[31] Iriel A., Bruneel S.P., Schenone N., Cirelli A.F. (2018). The removal of fluoride from aqueous solution by a lateritic soil adsorption: Kinetic and equilibrium studies. Ecotoxicol. Environ. Saf..

[32] Wang J., Chen N., Feng C., Li M. (2018). Performance and mechanism of fluoride adsorption from groundwater by lanthanum-modified pomelo peel biochar. Environ. Sci. Pollut. Res..

[33] Singh K., Lataye D.H., Wasewar K.L. (2016). Removal of Fluoride from Aqueous Solution by Using Low-Cost Sugarcane Bagasse: Kinetic Study and Equilibrium Isotherm Analyses. J. Hazard. Toxic Radioact. Waste.

[34] Raghav S., Kumar D. (2018). Adsorption Equilibrium, Kinetics, and Thermodynamic Studies of Fluoride Adsorbed by Tetrametallic Oxide Adsorbent. J. Chem. Eng. Data.

[35] Agrawal A., Sahu K.K. (2006). Kinetic and isotherm studies of cadmium adsorption on manganese nodule residue. J. Hazard. Mater..

[36] Oh T.-K., Choi B., Shinogi Y., Chikushi J. (2012). Effect of pH Conditions on Actual and Apparent Fluoride Adsorption by Biochar in Aqueous Phase. Water Air Soil Pollut..

[37] Huong P.T., Jitae K., Giang B.L., Nguyen T.D., Thang P.Q. (2019). Novel lanthanum-modified activated carbon derived from pine cone biomass as ecofriendly bio-sorbent for removal of phosphate and nitrate in wastewater. Rendiconti Lincei Sci. Fis. Nat..

[38] Wang Z., Shen D., Shen F., Li T. (2016). Phosphate adsorption on lanthanum loaded biochar. Chemosphere.

[39] Vences-Alvarez E., Velazquez-Jimenez L.H., Chazaro-Ruiz L.F., Diaz-Flores P.E., Rangel-Mendez J.R. (2015). Fluoride removal in water by a hybrid adsorbent lanthanum–carbon. J. Colloid Interface Sci..

[40] Yu Y., Yu L., Paul Chen J. (2015). Adsorption of fluoride by Fe–Mg–La triple-metal composite: Adsorbent preparation, illustration of performance and study of mechanisms. Chem. Eng. J..

[41] Ashraf I., Li R., Chen B., Al-Ansari N., Rizwan Aslam M., Altaf A.R., Elbeltagi A. (2022). Nanoarchitectonics and Kinetics Insights into Fluoride Removal from Drinking Water Using Magnetic Tea Biochar. Int. J. Environ. Res. Public Health.

[42] Jia Y., Zhu B.-S., Jin Z., Sun B., Luo T., Yu X.-Y., Kong L.-T., Liu J.-H. (2015). Fluoride removal mechanism of bayerite/boehmite nanocomposites: Roles of the surface hydroxyl groups and the nitrate anions. J. Colloid Interface Sci..

[43] Aghazadeh M., Golikand A.N., Ghaemi M., Yousefi T. (2011). A novel lanthanum hydroxide nanostructure prepared by cathodic electrodeposition. Mater. Lett..

[44] Qiu H., Liang C., Yu J., Zhang Q., Song M., Chen F. (2017). Preferable phosphate sequestration by nano-La(III) (hydr)oxides modified wheat straw with excellent properties in regeneration. Chem. Eng. J..

